# Functional mapping of the somatosensory cortex using noninvasive fMRI and touch in awake dogs

**DOI:** 10.1007/s00429-024-02798-0

**Published:** 2024-04-20

**Authors:** C-N Alexandrina Guran, Magdalena Boch, Ronald Sladky, Lucrezia Lonardo, Sabrina Karl, Ludwig Huber, Claus Lamm

**Affiliations:** 1 Social, Cognitive and Affective Neuroscience Unit, Department of Cognition, Emotion, and Methods in Psychology, Faculty of Psychology, University of Vienna, Vienna, Austria; 2 Vienna Cognitive Science Hub, University of Vienna, Vienna, Austria; 3 Comparative Cognition, Messerli Research Institute, University of Veterinary Medicine Vienna, Medical University of Vienna and University of Vienna, Vienna, Austria

**Keywords:** Somatosensation, Dog, fMRI, Touch, Higher-order cognition

## Abstract

Dogs are increasingly used as a model for neuroscience due to their ability to undergo functional MRI fully awake and unrestrained, after extensive behavioral training. Still, we know rather little about dogs’ basic functional neuroanatomy, including how basic perceptual and motor functions are localized in their brains. This is a major shortcoming in interpreting activations obtained in dog fMRI. The aim of this preregistered study was to localize areas associated with somato-sensory processing. To this end, we touched *N* = 22 dogs undergoing fMRI scanning on their left and right flanks using a wooden rod. We identified activation in anatomically defined primary and secondary somatosensory areas (SI and SII), lateralized to the contralateral hemisphere depending on the side of touch, and importantly also activation beyond SI and SII, in the cingulate cortex, right cerebellum and vermis, and the sylvian gyri. These activations may partly relate to motor control (cerebellum, cingulate), but also potentially to higher-order cognitive processing of somatosensory stimuli (rostral sylvian gyri), and the affective aspects of the stimulation (cingulate). We also found evidence for individual side biases in a vast majority of dogs in our sample, pointing at functional lateralization of somatosensory processing. These findings not only provide further evidence that fMRI is suited to localize neuro-cognitive processing in dogs, but also expand our understanding of in vivo touch processing in mammals, beyond classically defined primary and secondary somatosensory cortices.

## Introduction

In recent years, canine neuroimaging has seen a rise in interest (Berns et al. 2012; Bunford et al. 2017; Huber and Lamm 2017). The dog (*canis lupus familiaris*) as a model organism provides us with interesting features, such as similar living conditions as humans (ManyDogs Project et al., 2023) and a remarkable social cognitive ability (Hare and Tomasello 2005; ManyDogs Project et al., 2023; Topál et al. 2009).

To understand how higher order cognition is processed in the dog´s brain, we also need to understand the brain´s fundamental organization and how it processes sensory input at the lower levels. To this end, recent research has mapped out the visual, olfactory and auditory cortices in awake and unrestrained dogs using functional magnetic resonance imaging (fMRI; Andics et al. 2014, 2016; Boch et al. 2021, 2023; Bunford et al. 2020; Cuaya et al. 2016, 2022; Dilks et al. 2015; Gillette et al. 2022; Jia et al. 2014; Phillips et al. 2022). However, our best understanding of the canine somatosensory cortex dates back to almost 70 years ago (Fritsch & Hitzig, 1870/1963; Hamuy et al. 1956), using invasive methods, a small sample and focusing on selected parts of the canine cortex. Invasive methods have limitations in the investigation of the somatosensory cortex, even in humans (see Gordon et al. 2023, who found that a revision of the classic homunculus from Penfield and Boldrey 1937 was needed). Particularly whenever lesions are created, not only the welfare of the (animal) test subject is heavily disrupted, but lesions can additionally disrupt normal brain function, and therefore also disturb scientific results. In this study, we examined somatosensory processing in a sample of healthy and awake pet dogs. Our main aims were to understand how a non-primate mammal processes touch, whether their touch processing is lateralized, and finding out what parts of the brain may be involved beyond the primary and secondary somatosensory areas. With this approach, our study aimed to provide another important puzzle piece in comprehensively mapping and understanding dogs’ functional neuroanatomy in vivo and using non-invasive methodology.

During fMRI scanning, dogs were dynamically touched using a wooden rod moved down their left and right flanks. In line with our preregistration (https://osf.io/4gs9d/), we expected to see activation in the primary and secondary somatosensory cortices in response to touch (hypothesis 1), and that this activation would be higher in the hemisphere contralateral to the touched flank in primary somatosensory cortex SI, which in the dog is comprised of the postcruciate and rostral suprasylvian gyri, and in the ipsilateral hemisphere for secondary somatosensory cortex SII, the rostral ectosylvian gyri (Hamuy et al. 1956). Additionally, we hypothesized potential ipsilateral activation in the cerebellum, due to its (ipsilateral) involvement in motion control and potential involvement in suppressing touch elicited motion in our task (Uemura 2015). Importantly, since as of yet, our knowledge of canine brain function is still limited, we were also interested in uncovering additional brain areas related to in vivo touch processing. Hypothesis 2 focused on whether somatotopic mapping could be achieved with the relatively coarse temporal resolution of fMRI (Shmuel et al. 2007). In particular, we were interested in how activation shifts as a function of the dynamic somatosensory stimulation, possibly showing the trajectory of activation along the receptive fields coding for the parts of the back that were being stimulated, but also the progression of the signal into other parts of the canine cortex, to higher-order processing steps.

Moreover, we were interested in lateralization of somatosensory processing (hypothesis 3). While dogs may not possess a population wide side preference for one paw (Demirbas et al. 2023; Ocklenburg et al. 2019; Wells et al. 2018; but see Laverack et al. 2021), like humans favoring their right limbs (Papadatou-Pastou et al. 2020) or great apes do (Güntürkün et al. 2020; Hobaiter and Byrne 2013; Hopkins 2006), individually, most dogs seem to favor one over the other limb consistently (Ocklenburg et al. 2019). Even though somatosensation is not to be equated to motor behavior, we were interested in seeing whether somatosensation may be processed more strongly by one over another hemisphere within individuals (by looking at laterality quotients, hypothesis 3). Based on the existing literature for motor biases (Charlton and Frasnelli 2023; Ocklenburg et al. 2019; Simon et al. 2022), we expected the majority of dogs in our sample to show lateralized somatosensory processing.

## Methods

### Sample

Dogs in our sample had undergone extensive prior training to enable awake and unrestrained MRI scanning (Karl et al. 2020) and successfully participated in several prior studies (Boch et al. 2021, 2023; Guran et al. 2023; Karl et al. 2020b, 2021). For the present study, they additionally underwent familiarization with the touching rod and touching procedure. Dogs (and their owners) had been recruited through the Clever Dog Lab of the Messerli Research Institute at the University of Veterinary Medicine Vienna. Our final sample consisted of 22 dogs (9 female; mean age 4 years, age range: 1–8 years; mean weight = 21.9 kg). The sample consisted of a variety of fur types, as well as breeds, with 10 mixed-breed dogs, 5 retrievers, 6 shepherds, and 1 hunting dog. The touch stimulation delivered constant pressure, working similarly for dogs of different fur types. Our data collection stopping rule is specified in our analysis preregistration (https://osf.io/tdzrf), whereby we included all dogs that concluded two successful runs with less than 50% excluded volumes per run, by June 15th 2023.

All dogs were examined for their generally good health condition and eyesight at the Small Animals Clinics of the University of Veterinary Medicine Vienna prior to inclusion into the dog imaging cohort. Owners gave written informed consent before data collection but no monetary compensation was given to dog owners for their dog’s participation. This work was approved by the institutional ethics and animal welfare commission in accordance with Good Scientific Practice guidelines and national legislation at the University of Veterinary Medicine Vienna (ETK-06/06/2017), based on a pilot study conducted at the University of Vienna, and complies with the ARRIVE Guidelines (Kilkenny et al. 2010).

### Touch stimulation procedure

**Fig. 1:**
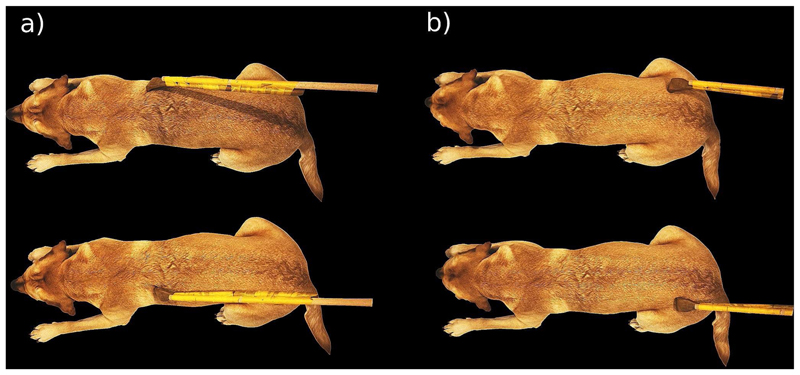
Illustration of the touching procedure and location. Touching started behind the left or right shoulder (**a**) and progressed for four seconds until reaching the hip area (**b**). Then, the other side was stimulated in the same way

Dogs lay on the scanner bed head first, in prone position, facing the back of the scanner. The dog trainers who performed the stimulation stood at the front of the scanner, to the right side of the scanner bed to touch the dogs with the rod. Trainers got auditory cues where to touch the dog, when to start, and when to stop, which were inaudible to the dogs. The rod was extended with another wooden stick to make sure that trainers did not need to reach into the scanner bore with their arms, which could potentially cause artefacts. We used a wooden backscratcher to touch the dogs on the right and left flanks, starting at the shoulder, all the way down to the hip joint (see Fig. 1). One dynamic stimulation lasted 4 s, and was repeated once (with a 1s break for the trainer to reposition the rod at the shoulder), before a baseline block (10 s). Then, the other side was touched. In each run, 10 blocks of stimulations were administered, 5 on each side (alternating). Each dog in this sample successfully underwent two full touch runs. Touch stimulations were blocked (10 s) and alternated with 10 s baselines (no touch).

### Touch training procedure

The dogs in our sample received extra training for the touch rod procedure. Most dogs (17 out of 22) only needed one training session, most of which took place at the mock scanner, while some needed between 2 and 5 training sessions. Trainings took roughly 20–45 min, depending on the dog, and varied in terms of training elements. In general, dogs were given the opportunity to explore the touch stick on their own first, after which motion of the stick was introduced by the trainer, touching the dog with the stick. As soon as this was tolerated well by the dog (as indicated by no signs of discomfort or avoidance behaviors), a step-by-step inclusion of the scanner elements followed, from performing touch by the rod while the dog was lying in the (mock) coil, on the scanner bed, being inside the (mock) scanner bore, and finally while playing back the scanner sounds while the dog was being touched with the rod.

### Data acquisition

Functional imaging data were obtained from 24 axial slices (interleaved acquisition in descending order, spanning the whole brain) using a twofold multiband-accelerated echo planar imaging (EPI) sequence with a voxel size of 1.5 × 1.5 × 2 mm^3^ (TR/TE = 1000/38 ms, Field of View (FoV) = 144 × 144 × 58 mm^3^, flip angle = 61°, 20% slice gap). The functional touch runs consisted of 215 volumes each. Structural images (134 volumes) were obtained using a voxel size of 0.7 mm isotropic (TR/TE = 2100/3.13 ms, FoV = 230 × 230 × 165 mm^3^). Runs could be acquired in the same or in separate sessions, depending on the dog’s capacity to lie still in the scanner for one or two runs in one session. We used a Siemens Magnetom Skyra (Siemens Healthcare, Erlangen, Germany) with a field strength of 3 Tesla for all measurements, and a head coil specifically designed for and tailored to optimal signal acquisition in dog fMRI (Guran et al. 2023).

### Data preprocessing

Data preprocessing was performed using SPM12 within MATLAB version 2022a. Images were slice-time corrected to the middle slice and realigned. Thereafter, we performed manual reorientation for the structural and EPI images. Structural images were manually skull-stripped with itk-SNAP (Yushkevich et al. 2006). This step is of particular importance in dog MRI, where the skull is bordered by massive musculature which can hinder successful coregistration, which was performed onto the mean image of each run. Structural segmentation of the brain was performed using the canine tissue probability maps provided by Nitzsche et al. (2019). Normalization of functional and structural data were performed using the “Old Normalization” module in SPM (originally implemented in SPM8), finally reslicing images to 1.5-mm isotropic voxel size, and smoothing of 3 mm (with a Gaussian FWHM kernel). Data were motion scrubbed by calculating framewise displacement, and excluding volumes with a displacement larger than 0.5 mm in comparison to the previous volume (Power et al. 2012, 2014). In our final sample, we excluded an average of 15.3% of volumes in each run (roughly 33 volumes). Note that as part of our overall quality assurance measures, runs with ≥ 50% of volumes exceeding this threshold were discarded, and repeated in another imagining session (until this criterion was fulfilled for two total runs per dog).

### Analyses

We used different analysis approaches based on our questions of interest. To localize somatosensory activation, we used mass-univariate whole-brain and regions of interest General linear model (GLM) analyses in SPM12 using a dog-tailored canonical hemodynamic response function (Boch et al. 2021). To test for somatotopic activation shifts as a function of time, we used a time-resolved finite impulse response (FIR) analysis.

#### GLM analysis

**Fig. 2:**
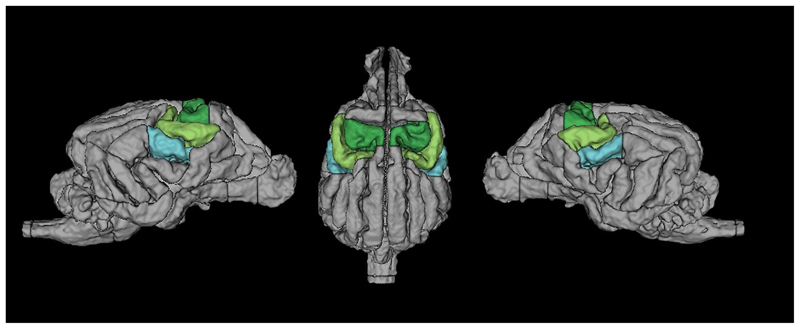
Location of SI in green, SII in light blue. Dark green = Postcruciate gyri. Light green = rostral suprasylvian gyri. Light Blue = rostral ectosylvian gyri (Czeibert et al. 2019)

We modelled the events of interest using a design matrix with the following regressors: Touch Left and Touch Right. From this we defined the following contrasts: Touch vs. Baseline (T vs. BL), which contrasted activation in response to left and right-sided touch to the baseline (no touch blocks, visual fixation), Right touch vs. Baseline (R vs. BL), where the right flank of the dog was touched, Left touch vs. Baseline (L vs. BL), where the left flank was touched. These contrasts were defined on a subject level (1st level) and then entered into a second level GLM analysis. In addition to a whole-brain exploration of activation in these contrasts, we tested *a priori* hypotheses on somatosensory engagement with higher sensitivity. To this end, we defined anatomical regions of interest (ROIs) for primary and secondary somatosensory cortex SI and SII, and used these as masks for an ROI analysis on the 2nd level, using the small volume family-wise error correction utility (SVC) in SPM12. Specifically, ROIs were based on previous invasive literature (Hamuy et al. 1956) and included the postcruciate and the rostral suprasylvian gyri as SI, and the rostral ectosylvian gyrus as SII, see Fig. 2.

Anatomical regions underlying clusters outside of the anatomical SI + SII mask refer to a dog anatomical atlas (Czeibert et al. 2019) which was normalized to a breed-averaged template space (Nitzsche et al. 2019). For statistically thresholding the whole-brain analyses, we had preregistered a cluster-level inference approach with a cluster-defining threshold of *p* < 0.005 (FWE-corrected at cluster level, α = 0.05) based on prior research; since the resulting clusters were unexpectedly large, we also used these criteria for the small-volume-correction analyses; note that this is a deviation from the preregistration (where we had preregistered *voxel-level* FWE-correction of α = 0.05).

#### FIR analysis

To ascertain whether “traveling” of activation across the cortex, in line with “caninculi” (in analogy to the creatures of the human somatosensory cortex, the homunculi) found in previous research (Penfield and Jasper 1954), or potential higher order processing, can be seen using fMRI while stimulating in a blocked design, we used a FIR analysis approach. We used bins of 1s each, thus splitting each stimulation (two per block) into four bins, and to visualize potential travel of activation across the stimulation. This analysis was performed separately for left and right touch stimulation. We compared the 1st and 4th (last) bin using T-tests. This contrast was chosen to maximize power for finding activation location differences. However, since we discovered no significant clusters in the fourth time bin, we also compared the first and third time bins. Finally, we compared each time bin to baseline levels and also inspected activation shifts across the time bins visually.

#### Lateralization analysis

To investigate lateralization of activation, we extracted beta values from right and left hemispheric anatomically defined unthresholded clusters in the T vs. BL contrast. We computed laterality quotients of beta weights per cluster, using this formula (see Seghier et al., 2008):

$$LQ=\frac{L_{mean\;}-R_{mean\;}}{\left|L_{mean\;}\right|+\left|R_{mean\;}\right|}$$

per dog. This formula puts the difference between activation in left and right hemispheric clusters in relation to the overall activation and deactivation in both hemispheres. We used a three factor solution for lateralization (Dragovic 2004), including a bin for side ambiguity. We set an arbitrary cutoff for considering dogs as lateralized if they had an LQ over 0.1 or under − 0.1 (a more conservative threshold than used elsewhere, see Zahnert et al., 2023; for a discussion of LQs see Seghier 2008). We took the absolute of all bias values and ran a t-test with them, testing against a 0 mean (no lateralization), to understand whether dogs´ brains are generally lateralized, irrespective of direction.

### Open science, data and code availability

We follow open science practices throughout, including pre-registration of the analyses, which can be found here: https://osf.io/tdzrf; canine fMRI data (2nd Level contrast SPM maps), as well as analysis scripts are available on github (https://github.com/alexandrinaguran/somatostudy).

## Results

### GLM results

#### Touch vs. baseline

To understand touch processing in the dog brain, we first ran t-contrasts in SPM using an anatomical mask of the SI and SII as an inclusive mask.

**Fig. 3:**
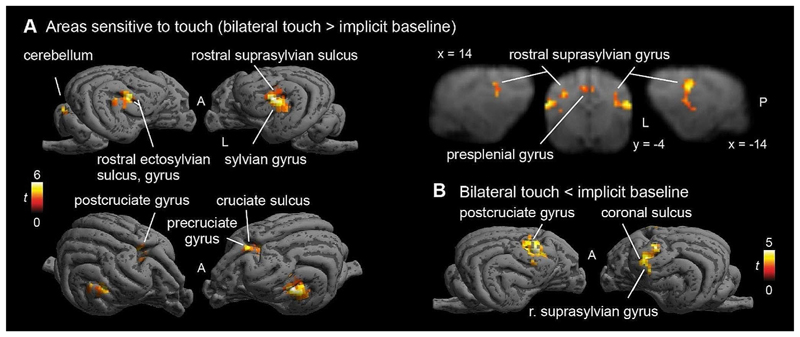
(**A**) Activations in response to Touch stimulation (whole-brain), *N* = 22. Bilateral SI and SII activation, as well as medial cluster (whole-brain), presumably in the cruciate sulcus and precruciate, as well as cingulate, gyrus. (**B**) Stronger responses in baseline than touch predominantly in postcruciate gyri. Image cluster thresholded at 0.005, 40 voxels

Contrasting Touch (bilateral) vs. Baseline, we found bilateral clusters located in the somatosensory cortex of the dogs, in a left hemispheric cluster (peak location: -20 -4 12) with k = 65, p_FWE_ < 0.001, T = 4.98, a right hemispheric cluster (peak location: 20 − 6 12) with k = 23, p_FWE_ < 0.05, T = 5.65, and a medial cluster (only at trend level, peak location: 2 0 19, k = 21, p_FWE_ = 0.053, T = 4.53), see Fig. 3A. There is a stronger activation in the touch condition, as opposed to the baseline condition. In the reverse contrasts (Baseline > Touch), investigating relative deactivation during the Touch vs. BL, we found two clusters, one right hemispheric (peak location: 12 2 19) with 105 voxels, p_FWE_ < 0.001, T = 5.26, and one left hemispheric (peak location: -16 2 14) with 63 voxels, p_FWE_ < 0.005, T = 5.05, see Fig. 3B (i.e. higher activation in the baseline condition than during Touch).

On the whole-brain level (without any anatomical or any other mask), all three cortical clusters reached highly significant levels (all p_FWE_ < 0.005), for both activations and deactivations. However, the clusters with stronger activation during Touch vs. BL showed strongly increased extent in the whole-brain analysis, e.g. more than twice the size for the first cluster, and even larger differences for the other clusters (see Table 1), and were located more posteriorly than the clusters in the reverse contrast (see Fig. 3B). In addition to the cortical clusters, we found a cerebellar cluster (peak location: 5–39 4, vermis) with k = 42, p_FWE_ = 0.038, T = 4.81, with stronger activation during Touch vs. BL.

#### Left-sided touch vs. baseline

**Fig. 4:**
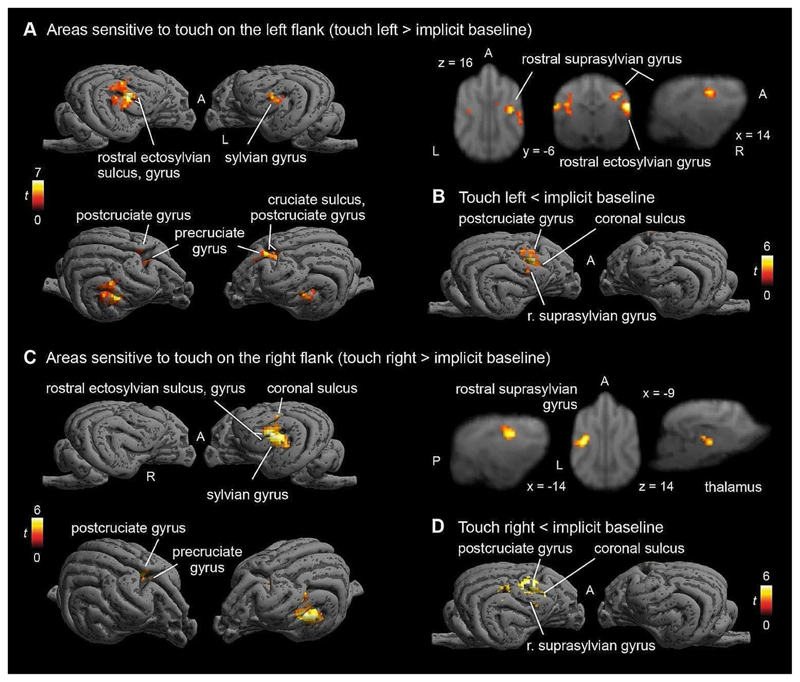
Blood oxygenetation level-dependent (BOLD) response to touch on left flank (**A+ B**) and right flank separately (**C + D**). Strong activation in posterior parts of SI and SII and rostromedial regions. De-activation in postcruciate gyrus (**B + D**). Image cluster thresholded at 0.005, 50 voxels. L, R, A, P: left, right, anterior, posterior, respectively

Contrasting touch on the left flank vs. Baseline, we found a large cluster in the right hemisphere (peak location: 20 − 6 12) with k = 74, p_FWE_ < 0.001, T = 6.9, as well as a smaller left hemispheric cluster, which only reached trend level in the SVC analysis (peak location: -16 -8 12) with k = 18, p_FWE_ = 0.073 (cluster level), T = 4.75, and a central cluster (peak location: 5 0 19) with k = 30, p_FWE_ = 0.016, T = 4.68, which showed stronger activation during Left touch than BL. In the opposite direction, we found one right hemispheric cluster (peak location: 14 4 20) with k = 70, p_FWE_ < 0.001, T = 6.18, showing stronger activation in Baseline than Left touch, see Fig. 4).

On the whole-brain level, a similar picture as in the Touch vs. BL analysis arose: descriptively all clusters were larger with smaller p-values (see Table 2).

#### Right vs. baseline

Contrasting touch on the Right flank with Baseline, we found a large cluster in the left hemisphere (peak location: -19 -4 12) with k = 83, p_FWE_ < 0.001, T = 5.73, with stronger activation for touch on the Right flank vs. Baseline (see Fig. 4C). In the opposite direction, we found one right hemispheric cluster (peak location: 12 2 18) with k = 79, p_FWE_ < 0.001, T = 5.97, with stronger deactivation during Right flank touch, see Fig. 4D.

Again, on the whole-brain level, we replicated all clusters we found in the SVC analysis, with larger cluster sizes.

Additionally, we found a central cluster as well as a left hemispheric cluster (see Table 3).

#### Investigating activation outside of anatomical masks

**Fig. 5:**
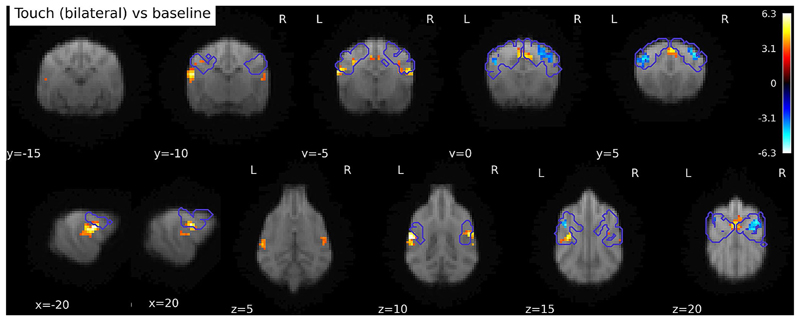
Activation in Touch vs. Baseline contrast, whole-brain. Purple contours: somatosensory cortex mask bounds (SI + SII). Clusters outside of SI + SII are ventral and caudal, as well as central. Image cluster thresholded at 0.005, 40 voxels

Through the GLM analysis and the notably larger clusters in the whole-brain analysis, it is apparent that the overlap between the observed activation and the regions we expected to be activated (as included in our anatomical masks) was not optimal. Therefore, we ran an exploratory SVC analysis using our anatomical mask as an *exclusive* mask, purely to better understand and illustrate the location of the activation outside of the anatomical mask. We found 4 significant clusters, one left hemispheric cluster, located close under SII, p_FWE_ = 0.001, k = 84, T = 6.27, peak location at -22 -8 10, one right hemispheric cluster, p_FWE_ = 0.038, k = 42, T = 5.16, peak location at 20 − 6 10. Additionally, we found one rostro-central cluster, p_FWE_ < 0.005, k = 73, T = 4.91, peak location at 0 4 22, and a cerebellar cluster, p_FWE_ = 0.038, k = 42, T = 4.87, at 5–39 4 (all p from cluster level inference). There were no significant clusters in the reverse contrast (BL vs. Touch). The left and right hemispheric clusters were located in the rostral and caudal sylvian gyri (left and right respectively), the central cluster comprised parts of the right cingulate gyrus and right precruciate gyrus. The cerebellar cluster was located in the right hemisphere and parts of the (right) vermis. Figure 5 shows all activations and deactivations in the main contrast (Touch vs. BL) with the contours of the anatomical mask superimposed. Please note that this analysis was not preregistered. We used the same inference levels as for all other analyses.

### FIR analysis

#### First vs. last time bins

**Fig. 6:**
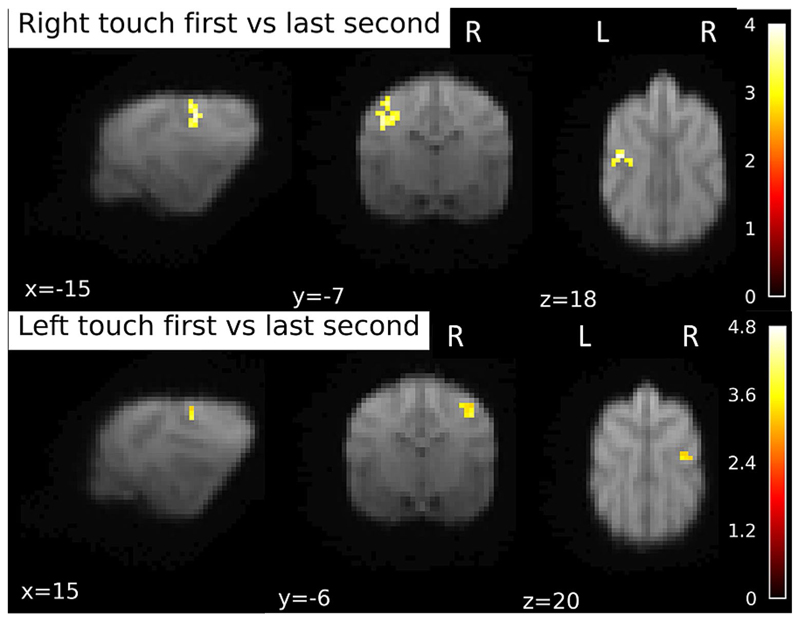
BOLD response during first second of touch stimulation contrasted to the last second of stimulation. Separately for right and left flank touch. Image of right touch cluster thresholded at 0.1 (to capture the trend level activations), 15 voxels. For left touch, 0.05 and 10 voxels

As above, we used an anatomical mask of SI and SII for SVC in these analyses, when not specified differently. Contrasting the first second of Touch stimulation on the right flank with the last (4th ) second of Right touch stimulation, we found only one cluster at trend level, p_FWE_ = 0.071, k = 35, T = 4.02, with peak location at -16 -6 18. This activation is significant when exploring the data using a mask that only included the contralateral left hemispheric SI and SII, with p_FWE_ < 0.005, see Fig. 6. There were no significant clusters in the opposite direction (4th time bin vs. first time bin). For the touch stimulation of the left flank, there was again a trend level cluster, p_FWE_ = 0.078, k = 17, T = 3.89, with the peak location at the exact contralateral side as found in the Right flank simulation, at 16 − 6 18. Again, using small-volume correction for the right hemispheric SI + SII, this cluster reached significance, p_FWE_ < 0.005. There were no significant clusters in the opposite direction (last time bin vs. first time bin). None of the clusters survived on the whole-brain level.

#### Time bins vs. Baseline

In addition to contrasting the two time bins where we expected the strongest difference (first and last), we looked at activation differences against Baseline for each time bin, on a whole-brain level. In particular, we were interested in seeing whether the activation visibly shifts across the somatosensory cortex as time progresses. For the first time bin (first second of stimulation), we found four clusters for the Left touch (two in the right hemisphere, one medial, one in the left hemisphere, see Table 4. All clusters survived the whole-brain level. For the first second of Right touch stimulation we found a large left-hemispheric cluster (k = 131), which not only survived on the whole-brain level, but was notably larger (k = 356), see Table 4. For the second and third time bins, there was at minimum one cluster in the hemisphere contralateral to the touch stimulation, which survived whole-brain level, see Tables 5 and 6. There were no significant clusters in the fourth (last) time bin.

#### Visual inspection of activation shift

**Fig. 7:**
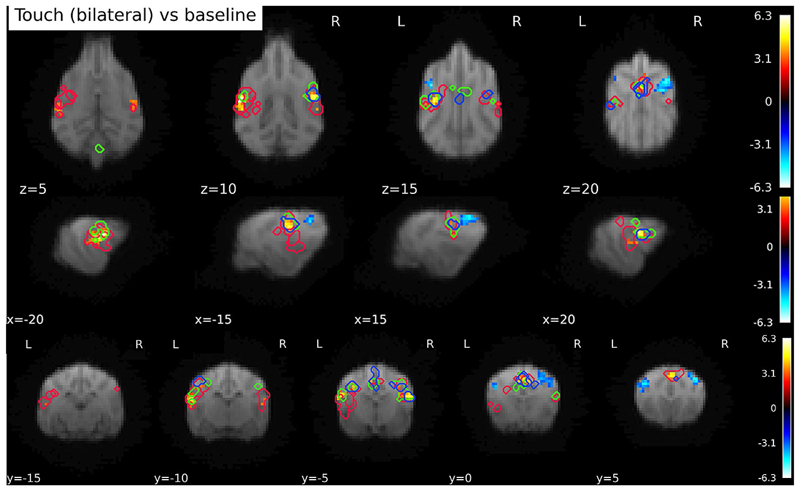
Clusters of activation by time bin, plotted on top of the Touch vs. BL contrast. Red outlines mark the activation in response to touch in the first time bin (0–1 s), green outlines activation in the second time bin (1–2 s), and blue the activation of the third time bin. No significant clusters were found in the fourth time bin. The figure illustrates both a relative overlap of activations, but also a shift towards more dorsal parts of SI and SII over the course of time

While statistical differences between time bins were observed, we also wanted to get an idea of the activation changes as a function of time. To this end we inspected data visually, by plotting the significant clusters found in each time bin onto the Touch vs. BL activation, see Fig. 7 below. From the visual inspections, the following qualitative observations seem to be possible: (1) activation is particularly strong in the first bin, and virtually nonexistent in the final bin. (2) Activation shifts to more central areas from the first to the third bin. (3) The peak of cerebellar activity happens during the second time bin. (4) Activations in the second and third time-bin seem more concentrated on central and dorsal areas, compared to activation during the first bin.

#### Comparison of first and third time-bin

Since there were no significantly activated clusters in the 4th time bin, we decided to run a comparison between the first and the third bin. Please note that this analysis was not preregistered or planned, but arose from the analysis of the data, i.e. the result that no significant clusters were found in the 4th time bin.

**Fig. 8:**
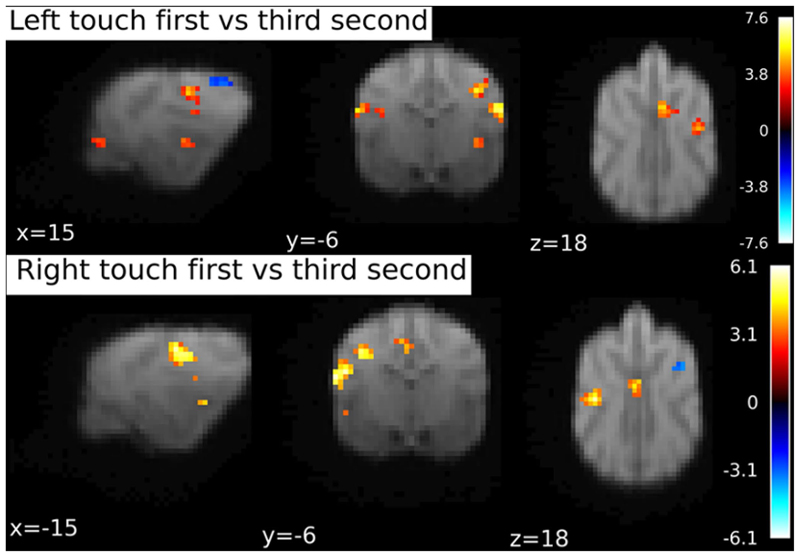
BOLD response during first second of touch stimulation contrasted to the third stimulation second. Separately for Right and Left flank touch. Image cluster thresholded at 0.05, 10 voxels

Comparing the first and third time bin on the left and right hemisphere separately, again using SVC with an anatomical mask of SI and SII, we found a left hemispheric cluster, p_FWE_ < 0.001, k = 94, T = 6.12 with peak at -19 -4 12 in response to Right flank touch, and a right hemispheric cluster, p_FWE_ < 0.001, k = 67, T = 7.63 with peak location at 20 − 4 12, and a rostro-central cluster, p_FWE_ = 0.02, k = 23, T = 4.75, peak location at 2 0 19, in response to Left flank touch, see Fig. 8.

### Laterality quotients

Out of the sample of 22 dogs, 19 showed a lateralization, 9 towards the right hemisphere and 10 towards the left hemisphere (values smaller than − 0.1 or bigger than 0.1 respectively). The mean absolute lateralization quotient was 0.49.

For the dogs whose touch processing was lateralized to the left hemisphere, the mean was 0.54 (± 0.36 std), and for the right-hemisphere-processing dogs 0.58 (± 0.4 std).

The sample showed a sample wide asymmetry, p_FWE_ < 0.001, T_21_ = 5.9 (undirected). This was also true for the left (*p* < 0.001, T_9_ = 4.71) and right (p_FWE_ < 0.005, T_8_ = -4.32) lateralized dogs separately.

**Fig. 9:**
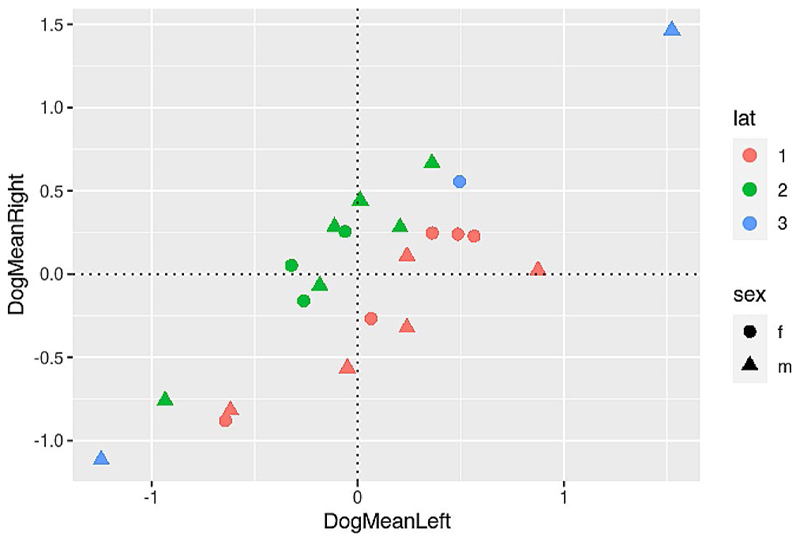
Relationship between mean left and right activation and laterality indices (with absolute values in the denominator), see 2.5.2. Circles represent female, triangles male dogs. Blue markers indicate no lateralization (values between − 0.1 and 0.1), green a rightward processing preference (LQ higher than 0.1), and red a leftward processing preference (lower than − 0.1)

Since there is some debate as to how to interpret negative mean beta weights, we plotted the left and right hemispheric cluster means per dog, see Fig. 9 including areas where LQ quotients above 0.1 and below − 0.1 can be found.

## Discussion

To understand touch processing in awake, healthy dogs, we touched dogs on their right and left flanks while undergoing non-invasive brain scanning using fMRI. Building on invasive work from the 1950s, we found activation (and deactivation) in areas previously identified as SI and SII of the dog, but also activation in other brain areas. We also found evidence for lateralization of responses and a shift of functional activity hotspots in line with the dynamics of the stimulation.

Firstly, we found clusters in the left and right hemisphere that responded more strongly to touch than baseline (no touch), as well as in a medial cluster. However, in addition to the hypothesized activation, we found additional areas outside the somatosensory areas that responded to touch stimulation. Touch activated the rostral and caudal sylvian gyri left and right, areas ventral and slightly caudal to SII, as well as the right cingulate cortex and right precruciate. The rostral sylvian gyrus has been linked to emotion perception (Hernandez et al. 2017; Karl et al. 2021), the detection of familiar vs. unfamiliar speech (Cuaya et al. 2022), or action observation (Boch et al. 2023), and therefore seems to act as a multisensory area that could play a role in sensory integration and association processes (see also Boch, Huber, et al. 2023 for review). Interpreting the findings, it seems unlikely that the areas outside of SI and SII would be directly related to primary sensory processing, based on findings in dogs but also other mammalian species, when it comes to touch processing. Rather, they might be related to higher-order processing of touch. In the case of the gyrus cinguli, affective processing of the touch stimulation may be localized there, as the gyrus cinguli has been shown to relate to affective and even social processes in both humans and non-human primates (Devinsky et al. 1995; Rudebeck et al. 2006; Vogt et al. 2005) but also to motor control (Wang et al. 2001). Little is known about the functionality of the canine cingulate cortex so far, but it may play a role in integration of information and functional processing (Szabó et al. 2023). Additionally, the central cluster was biased towards the right side. In dogs, different side biases for processing of sensory inputs exist, and namely, the right hemisphere may play a larger role in processing arousing (or negative) stimuli (Simon et al. 2022; Siniscalchi et al. 2017). Through the domestication process and intensive handling by humans, experienced by most dogs, it is conceivable that their brains show particular adaptations to process touch administered by humans in a social way, which may be linked to increased arousal. Finding involvement of higher order cognitive processes highlights the methodological advantage of using a non-invasive method in healthy functioning animals: these findings can be made impossible when other parts of the brain are removed or damaged surgically (Hamuy et al. 1956; Marshall et al. 1937).

When contrasting left or right flank touch separately to baseline, we did find major activation in the contralateral hemispheres in locations consistent with SI/SII (rostral suprasylvian and ectosylvian gyri right, left suprasylvian gyrus). Ipsilateral processing was only found for the left touch, and also only at trend level in the SVC analysis, but remained absent for right flank touch. Peak locations of activations were almost identical for both hemispheres. These results suggest a strong preference for contralateral processing and potentially a bigger role of SI in flank stimulation, in line with previous findings on touch processing in mammals (e.g. Catania and Remple 2002; Santiago et al. 2007; Welker 1976).

We also found cerebellar activation, particularly in the vermis, which is generally associated with motor control and conditioning (Glickstein and Yeo 1990; Ohyama et al. 2003): both aspects may have been relevant here, as the dogs are both relying on their learned behavior to lie still in the scanner and not respond with an orienting response to the touch stimulation, but also need to actively suppress motor responses. This is also in line with the observed deactivation in the postcruciate gyrus (rostral SI right). Interestingly, we found the deactivation only in the right hemisphere, for both directions of touch. Due to its proximity to areas that have been suggested to play a role in motion (precruciate gyrus), it may be that the right postcruciate gyrus stands in close connection to downregulation of precruciate activity, playing a role in the inhibition of motor responses (but see Cook et al. 2016, which showed frontal areas involved in inhibition, however no precruciate activity).

Our second hypothesis related to the possibility of tracking activation shifts across the 4s stimulation period starting at the shoulder and ending at the hip. Contrasting the first and third time bin, we found activation in the left and right hemispheres as well as a rostro-central cluster, aligning with parts of the postcruciate gyrus left and right: Importantly with time, processing moved away from the primary sensory areas and towards potentially higher-order association related parts of the cortex (see Fig. 7), as revealed by higher engagement of rostro-central cortical areas. Thus, the FIR analysis approach allows some insights into the progression of sensory signal processing across higher-order cortical areas (i.e., outside the primary and secondary somatosensory cortices). However, possibly due to the lower resolution representation of the back (see e.g. Gordon et al., 2020), and an ebbing off of activity after the initial touch in the primary cortex, other touch procedures (different start points and different body parts) should be considered by future studies. This will also be important for mapping caninculi in a more comprehensive fashion. Note that one reason we had chosen to stimulate the back of the dogs in this first exploratory study on somatosensory responses were the feasibility of stimulation and their tolerance by the dogs in the challenging MRI environment.

Finally, our third aim was to investigate whether dogs show lateralization (hemispheric dominance) of somatosensory processing. Already in the GLM analysis, we found some effects that indicated substantial lateralization, such as the deactivation in the right postcruciate gyrus. While somatosensory processing should not be equated with lateralized motor preferences, such as pawedness, which are more commonly investigated, lateralization in somatosensory processing can be related to motor biases and could be indicative of a generalized feature shared by many vertebrates (Güntürkün et al. 2020; Rogers et al. 2013). Roughly 86% of dogs (19 out of 22) showed a lateralization bias towards one hemisphere, as quantified through beta weights, a significant deviation from 0, with roughly equal numbers for left and right hemispheric biases. While there is no population wide preference for use of the left or right paw, around 70% of dogs generally show an individual side preference. This number is lower, but in a similar ballpark, than the number of dogs that showed a lateralized processing in our sample here. Thus, sensory processing is lateralized in most dogs. These data give no reason to assume a population wide side bias of processing, in contrast to a left-ward grey matter volume bias in dogs in general (Barton et al. 2023), suggesting a complex relation between functional and structural asymmetries.

Canine fMRI research is often limited by its sample sizes, an issue we circumvented by extending data collection until reaching a sample of 22 dogs, however, the future of canine and comparative neuroscience should be collaborative, to maximize power for important questions on canine cognition and cognitive evolution (ManyDogs Project et al., 2023). Our main interest lay in the identification of touch elicited cortical activation against baseline, a very strong effect for which we deemed our sample size to be more than sufficient. However, statistical power with the current sample size may have been suboptimal for some analyses, but in particular the FIR analysis which comes with a number of statistical tests that need to be corrected for (thus decreasing power; see also Huber and Lamm 2017, for further discussion regarding issues of power and sensitivity in dog fMRI research).

In our approach of touch stimulation, a few limitations should be noted: first of all, the stimulations were performed by the dog trainers, and not a robotic apparatus. While timings and the procedure itself were both practiced and cued, it is very likely that a degree of variability in stimulations was present, which is of relevance to the FIR analysis, in addition to the coarse neural somatosensory representation of the back in comparison to other areas (Coq et al. 2004; Kaas 1993; Woolsey et al. 1942). We chose this area pragmatically: stimulating the dog from the front would have brought the trainer into the view of the dog, possibly inducing distraction and other effects. From the back of the scanner, only the flanks and the paws were accessible, and many dogs do not like being touched on their paws and show this by moving them out of reach. Thus, only the back stimulation was viable. However, even though the back may not be the somatosensorily best represented area of the dog´s body, we were still able to find differences between touch bins on this area. Finally, for the analysis of lateralization, within fMRI research, it is a continuing debate how to proceed with negative values. Since no consensus exists in the literature, one can a) look at negative and positive values separately, or b) use absolute values in the denominator of the fraction used to calculate laterality indeces (Seghier 2008). We opted for the latter version, since we would have shrunk the sample if only looking at either negative or positive values (not all dogs *had* both positive and negative values). The accuracy of activation localizations in fMRI is always limited by the signal that can be collected. While we were able to collect data at a (relatively high) resolution of 1.5 × 1.5. x 2 mm^3^ functionally, dogs´ brains are also quite small, and vary in shape depending on breed. However, our localizations are based on relatively large anatomical regions of interest (see Fig. 2), within a small range of dog sizes and relatively uniform breeds (all dogs were mesaticephalic). We thus suggest that the spatial resolution was well-matched to detect our main areas of *a priori* interest.

In general, however, we want to point out the strengths of (f)MRI in the investigation of non-human cognition. While fMRI may seem limited to humans, or highly trainable species, advances have been made with a broad range of animals, from farm animals (Pluchot et al. 2023), to crocodiles (Behroozi et al. 2018), and birds (Behroozi et al., 2019; DeGroof et al. 2013). MRI allows us to not only investigate the overall structural anatomy of brains, but also their function in a non-invasive manner. This not only comes at the benefit of animal welfare, making sure the animal can continue living healthy lives, in this case with their human caregivers, but also enhances the bandwidth of possible findings: Especially links to higher-order processing areas that may be unexpected can be hindered by invasive studies, which require pathways and areas to be partly lesioned, or which only allow to target specific pre-selected brain areas (while MRI allows assessment of the whole brain at once). Thus, using fMRI, it is possible to investigate not only lateralization, but most importantly, higher-order processing of the intact brain.

Using fMRI, we confirmed the roles of the dog’s postcruciate and rostral suprasylvian gyri (SI) as well as the rostral ectosylvian gyri (SII) in the processing of touch. Beyond these areas that are likely most closely tied to sensory-perceptual computation, we pinpointed additional areas, namely parts of the rostral cingulate cortex, which may relate to affective processing, as well as the sylvian gyri, which may play a putative role in higher-order or associative somatosensory processing. We found somatosensory processing to be lateralized in most dogs, with the sample being evenly split between left-, and right-hemisphere biased dogs. First indications for a somatotopic organization based on analyses of activation dynamics need further corroboration. Our findings add another puzzle piece in the dogs’ functional neuroanatomy and will be helpful in charting brain function across diverse species.

## Figures and Tables

**Table 1:** Significant clusters in the wholebrain analysis, contrasting Touch (both sides) with Baseline. Clusters are similar to those found in the SVC (see peak locations), however much larger and have generally higher T-values and better significance

Contrast	p (FWE-corrected)	size (k voxels)	T	peak location	Anatomical label of peak
Touch vs.	< 0.001	171	6.27	-22 -8 10	Rostro-dorsal ectosylvian g. left
BL	0.002	74	5.65	20 – 6 12	Rostral suprasylvian g. right
	< 0.001	110	4.91	0 4 22	Gyrus cinguli
	0.038	42	4.87	5–39 4	Vermis (cerebellum) right
BL vs.	< 0.001	125	5.26	12 2 19	postcruciate gyrus right
Touch	0.001	77	5.05	-16 2 14	postcruciate gyrus left

**Table 2:** Significant clusters in the whole-brain analysis of left flank touch vs. baseline

Contrasts	p (FWE-corrected)	size (k voxels)	T	peak location	Anatomical label of peak
Left vs. BL	< 0.001	154	6.93	22 – 4 10	Sulc. Between ectosylvian and sylvian gyr. right
	0.008	56	4.93	-22 -6 10	Sulc. Between ectosylvian and sylvian gyr. left
	< 0.001	113	4.86	2 8 22	Cingulate gyrus right
BL vs. Touch	0.001	81	6.18	14 4 20	Postcruciate gyrus right

**Table 3:** Significant clusters in the whole-brain analysis, contrasting Right touch and Baseline. Note the additional central and left hemispheric clusters, absent in the SVC analysis

Contrasts	p (FWE-corrected)	size (k voxels)	T	peak location	Anatomical label of peak
Right vs. BL	< 0.001	191	5.84	-20 -12 7	Suprasylvian sulcus left
	0.013	53	4.96	-2 0 19	Cingulate left
	0.013	53	4.47	-6 -9 1	
BL vs. Right	< 0.001	135	5.97	12 2 18	Postcruciate sulcus(?)

**Table 4:** Significant clusters contrasting the first time bin against Baseline on a whole-brain level

Touched Flank	p (FWE-corrected)	size (k voxels)	T	peak location
Left	< 0.001	109	8.31	22 – 4 10
	0.005	50	7.2	16 – 6 16
	< 0.001	126	5.42	-19 -10 13
	< 0.001	101	4.62	0 6 24
Right	< 0.001	356	6.57	-20 -3 10

**Table 5:** Significant clusters contrasting the second time bin against Baseline on a whole-brain level

Touched flank	p (FWE-corrected)	size (k voxels)	T	peak location
Left	0.001	78	6.13	18 – 4 12
Right	0.001	71	5.42	2–2 16
	< 0.001	98	5.2	-19 -9 16
	0.043	38	3.75	2–34 2

**Table 6:** Significant clusters contrasting the third time bin against Baseline on a whole-brain level

Touched Flank	p (FWE-corrected)	size (k voxels)	T	peak location
Left	< 0.001	96	5.97	2 0 19
Right	0.008	53	6.34	-13 -4 16
	0.001	72	3.99	-1 0 19

## Significance

To understand brain function and evolution, it is necessary to look beyond the human lineage. This study provides insights into the engagement of brain areas related to somatosensation using whole-brain non-invasive neuroimaging of trained, non-sedated, and unrestrained pet dogs. It showcases again the usefulness of non-invasive methods, in particular fMRI, for investigating higher-order brain function and advances the mapping of brain functions in dogs; using this non-invasive approach without sedation, we are able to identify previously unknown potential higher-order processing areas and offer a quantification of touch processing lateralization.
